# Public private partnership in in-service training of physicians: the millennium development goal 6-partnership for African clinical training (M-PACT) approach

**DOI:** 10.11604/pamj.2018.29.77.14480

**Published:** 2018-01-25

**Authors:** Obinna Ositadimma Oleribe, Babatunde Lawal Salako, Albert Akpalu, Emmanuel Anteyi, Mamadou Mourtalla Ka, Gibrilla Deen, Temilola Akande, Mei Ran Abellona U, Maud Lemoine, Mairi McConnochie, Matthew Foster, Richard Walker, Simon David Taylor-Robinson, Ali Jawad

**Affiliations:** 1 Excellence and Friends Management Care Centre (EFMC), Dutse Abuja FCT, Nigeria; 2 Royal College of Physicians of London, 11 St Andrews Place, Regent’s Park, London NW1 4LE, United Kingdom; 3 West African College of Physicians (WACP), 6 Taylor Drive, Off Edmond Crescent, Yaba Lagos Nigeria; 4 Department of Medicine, College of Medicine, University of Ibadan and University College Hospital, Ibadan, Oyo State, Nigeria; 5 Korle Bu Teaching Hospital, Accra, Ghana; 6 National Hospital, Abuja, Nigeria; 7 University of Thiès, Region of Thiès, Senegal; 8 University of Sierra Leone Teaching Hospitals Complex-Connaught Hospital, Freetown, Senegal; 9 Department of Surgery and Cancer, Imperial College London, Exhibition Road, London SW7 2AZ, United Kingdom; 10 Department of Medicine, North Tyneside General Hospital, Tyne & Wear, NE29 8NH, United Kingdom; 11 Hepatology Unit, Imperial College London, 10^th^ Floor, QEQM Building, St Mary’s Hospital Campus, South Wharf Road, W2 1NY, London, United Kingdom

**Keywords:** Continuous professional development, public-private partnership, postgraduate medical education, in-service training

## Abstract

**Introduction:**

in-service training of healthcare workers is essential for improving healthcare services and outcome.

**Methods:**

The Millennium Development Goal (MDG) 6 Partnership for African Clinical Training (M-PACT) program was an innovative in-service training approach designed and implemented by the Royal College of Physicians (RCP) and West African College of Physicians (WACP) with funding from Eco Bank Foundation. The goal was to develop sustainable capacity to tackle MDG 6 targets in West Africa through better postgraduate medical education. Five training centres were establised: Nigeria (Abuja, Ibadan), Ghana (Accra), Senegal (Dakar) and Sierra Leone (Freetown) for training 681 physicians from across West Africa. A curriculum jointly designed by the RCP-WACP team was used to deliver biannual 5-day training courses over a 3-year period.

**Results:**

Of 602 trained in clinical medicine, 358 (59.5%) were males and 535 (88.9%) were from hosting countries. 472 (78.4%) of participants received travel bursaries to participate, while 318 (52.8%) were residents in Internal Medicine in the respective institutions. Accra had the highest number of participants (29.7%) followed by Ibadan, (28.7%), Dakar, (24.9%), Abuja, (11.0%) and Freetown, (5.6%). Pre-course clinical knowledge scores ranged from 35.1% in the Freetown Course to 63.8% in Accra Course 1; whereas post-course scores ranged from 50.5% in the Freetown course to 73.8% in Accra course 1.

**Conclusion:**

M-PACT made a positive impact to quality and outcome of healthcare services in the region and is a model for continued improvement for healthcare outcomes, e.g malaria, HIV and TB incidence and mortality in West Africa.

## Introduction

There are serious disparities in the distribution, specialization, diversity and competency of the healthcare workforce across the world. Countries with fewer healthcare professionals have poorer health outcomes, compared to those that have a larger number of better trained individuals [1]. Moreover, medicine is changing at a striking speed, making in-service training of available healthcare workers, especially physicians, a must for better healthcare services, improved healthcare outcomes and reduced rates of hospital-associated morbidity and mortality. Thus, in-service training of physicians has become an established requirement in many countries and is common among physicians studying for postgraduate medical qualifications around the world. However, when medical careers are not linked to postgraduate medical degrees and diplomas, or to governmental revalidation requirements for medical licensing, physicians in many countries often fail to attend continuous professional development training. Outside the remit of postgraduate degrees, annual licensing and compulsory update courses, there are several areas of postgraduate development to which physicians ought to be exposed regularly in order to improve healthcare outcomes in sub-Saharan Africa. In the West African context, these include training in leadership, project management, infectious disease outbreak investigations and advances in important diseases in the sub-region, such as malaria, HIV/AIDs and tuberculosis. Recognizing the need to train physicians in these areas, the West African College of Physicians (WACP) and the Royal College of Physicians of London (RCP) partnered to raise funds from non-traditional sources to support physicians from West Africa to attend specific training courses on these topics. In-service training has been shown to improve physician rating and patient satisfaction [2, 3]. However, in-service training represents a significant financial governmental investment for supporting continued competence of the healthcare workforce in West Africa [4]. On the other hand, decreasing global resources are a critical limitation to increased access to in-service training, particularly as income from the petrochemical industry, that might have funded such projects in the past, is at a relative nadir currently in West Africa.

Thus, there is need to identify sustainable funding mechanisms towards regular and continuing in-service training of physicians in the West African region. This is all the more critical in the face of a pervasive and long-term shortage of skilled healthcare workers in the region. Previous studies have revealed that case-based learning, clinical simulations, practice and feedback are effective educational techniques; and that didactic techniques that involve passive instruction have little or no impact on learning outcomes [4]. The studies also stated that superior learning outcomes result from mentorship provided to participants [4]. However, as gains from in-service training are usually short-lived [3], it is advisable that participants are monitored over a period of time. As public funds are usually inadequate for the pre-and in-service training of healthcare workers, there is a need to secure funding from the private sector to support the development and improvement of the health infrastructure. Over the past few years, the RCP has had a progressive partnership with the WACP, resulting in both training of physicians and development of various training curricula. In 2013, these two medical postgraduate organisations developed a new project, the Millennium Development Goal (MDG) 6-Partnership for African Clinical Training (M-PACT). The goal of M-PACT was to develop sustainable capacity to tackle MDG 6 targets-to combat tuberculosis, HIV/AIDS, malaria and other communicable diseases, in West Africa through better postgraduate medical education in malaria, HIV/AIDS and tuberculosis, together with honing medical and organisational skills in disease outbreak management, such as in Ebola virus disease, which was particularly topical in West Africa at that time. The project aimed at establishing five Millennium Development Goal 6 Training Centres (MTCs) in Abuja, Accra, Dakar, Freetown and Ibadan to deliver sustainable education and training for doctors across the region to improve patient care. The original MDG 6 goals aimed to have halted the rising incidence of tuberculosis, HIV/AIDS and malaria in West Africa by 2015 and to have begun to reverse the spread of HIV/AIDS while achieving universal access to treatment for HIV/AIDS by 2010 for all those who needed it [5]. Unfortunately, these goals were not achieved and hence this highlights the importance and significance of the M-PACT project in developing sustainable postgraduate training in these medical areas of expertise in order to combat the problems more effectively. The goal of this article is to share an innovative 3-year in-service training approach designed and implemented by the RCP in partnership with the WACP with funding from Eco Bank Foundation (Lomé, Togo).

## Methods

**Figure 1:**
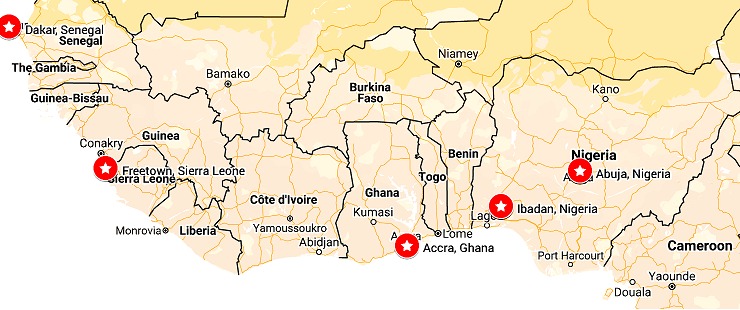
Locations of RCP-WACP M-PACT training centers in West Africa, 2014-2017

The RCP and WACP initiated the M-PACT program with funding obtained from Eco Bank Foundation (Lomé, Togo) to train 681 physicians from across the West African region in the postgraduate skills of medical leadership, malaria, tuberculosis and HIV/AIDS. We established five training centres in Nigeria (Abuja and Ibadan), Ghana (Accra), Senegal (Dakar) and Sierra Leone (Freetown) (Figure 1). Following the establishment of training centres of excellence in the four nations, we set up a project board drawn from RCP, WACP, partner institutions and Eco Bank Foundation. Two project managers were employed for London and West Africa to coordinate medical training experts, who volunteered from the United Kingdom (through the auspices of the RCP) and to arrange the courses in West Africa over a 3-year period. The RCP-WACP team finalized the training curricula over a 3-day period in London, United Kingdom. We engaged RCP fellows as volunteers, and worked with national conveners as the key contact persons in each centre. Each convener developed their own administrative support and engaged local faculty to deliver the courses. The training curricula were slightly different at each location depending on local needs, with Nigerian centres focusing on renal complications, Ghana on neurological and cardio-pulmonary complications, Senegal on gastrointestinal and dermatological complications and Sierra Leone on renal and dermatological complications of HIV/AIDS, TB and malaria. We openly advertised the various courses and suitable applicants were enrolled into the course. Faculty members developed courseware and these were reviewed by senior peer reviewers to ensure quality was met and sustained. Training was for a period of 5 days with a day each focusing on malaria, TB, HIV and medical leadership skills, infectious disease outbreak investigation, and community care were also covered. We used the early mornings to refresh participants' understanding of the epidemiology, pathophysiology of and recent advances in each disease area. The early afternoons were used for workshops on the various national programs and organ/system complications of the various diseases. The evening periods were used for participant presentation, mentoring and bedside teaching. We used pre-and post-course testing, as well as daily and summative assessment to evaluate the courses. We also conducted a six-month post-training evaluation. Project managers with key volunteers, national conveners and board members held annual review meetings to assess the quality and outcome of the training. We evaluated and implemented suggestions from participants and ensured that weaknesses were mitigated either immediately or during subsequent courses. We translated courseware into French to cater for the Francophone countries and participants who could not speak English. As educational techniques are critical to learning outcomes and repetitive interventions can result in better learning outcomes [4], we trained faculty first on facilitating and teaching techniques using the Doctors as Educators program curriculum.

## Results

**Figure 2:**
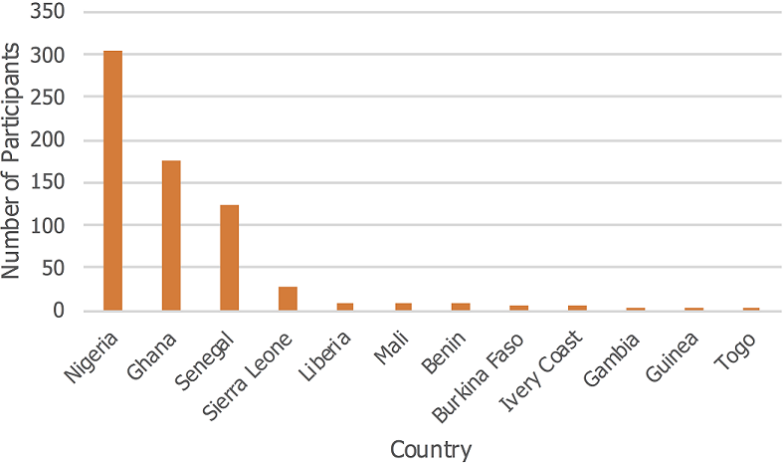
Participants of RCP-WACP M-PACT program drawn from 12 nations of West Africa 2014–2017

**Figure 3:**
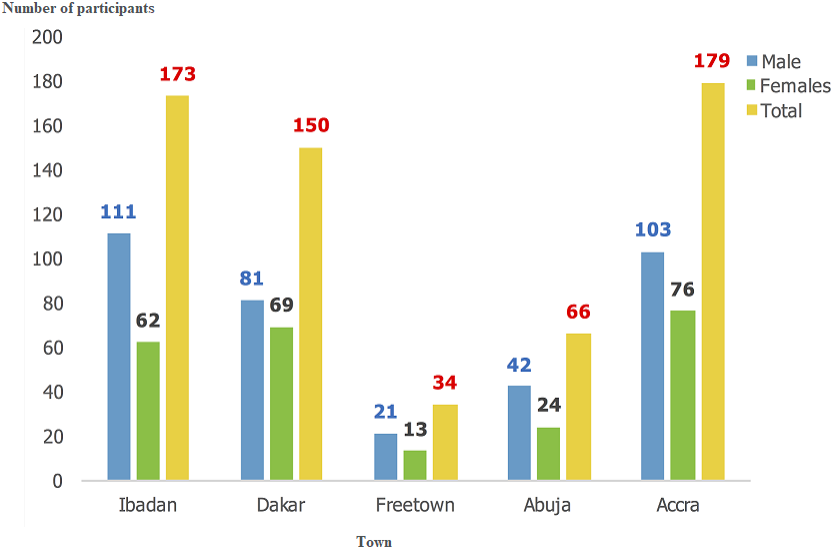
Sex and location distribution of participants of the M-PACT courses in five centres

**Figure 4:**
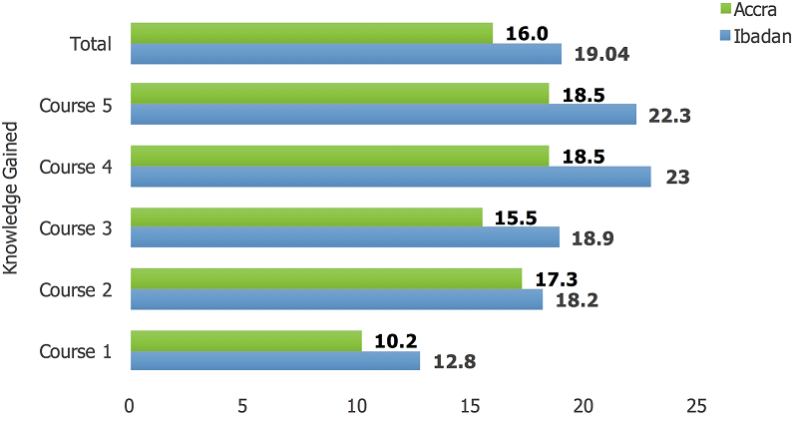
Knowledge gained from the M-PACT course in Accra, Ghana vs Ibadan, Nigeria over the five courses, 2014–2017

A total of 18 one-week clinical training courses in five MDG 6 training centres were undertaken in Nigeria, Ghana, Senegal and Sierra Leone. Five courses were held in Ibadan, Accra and Dakar, two were held in Abuja and one in Freetown. Also, three one-week Doctors as Educators (DAE) workshops ("Training the Trainers") took place in Ibadan, Abuja and Accra. There was no DAE workshop in Dakar as the training of doctors in the nation had incorporated the DAE concepts. Also, in Freetown, this was not done as it was not economically efficient since only one course was held in the last year of the program. To mitigate this, faculties from both North and South were used to execute the Sierra Leone training. We trained a total of 703 doctors across the five centres, drawn from 12 West African countries of whom 101 (16.8%) were trained in the DAE courses (Figure 2). The majority of the participants were from Nigeria (45%), Ghana (26%) and Senegal (18%). Of the 602 trained in clinical medicine, 358 (59.5%) were males and 535 (88.9%) were from the local nations of Nigeria, Ghana, Senegal and Sierra Leone. Also, 472 (78.4%) of all participants received a travel bursary to participate in the course, while 318 (52.8%) were residents in Internal Medicine in their various institutions. Accra had the highest number of participants (29.7%) followed by Ibadan, (28.7%), Dakar, (24.9%), Abuja, (11.0%) and Freetown, (5.6%) as shown in Figure 3. Course evaluation showed a massive improvement between the pre- and post-course test with the largest improvement seen in Ibadan course 4 (Figure 4). Pre-course clinical knowledge scores ranged from 35.1% in the Freetown course to 63.8% in Accra course 1; while post-course clinical knowledge scores ranged from 50.5% in the Freetown course to 73.8% in Accra course 1. Knowledge gain was lowest for the Abuja course 2 at 9.0%, followed by Abuja course 1 (10.0%) and Accra course 1 (10.2%); but highest at Ibadan course 4 (23%), followed by Ibadan course 5 (22.3%). In addition, Ibadan had an average knowledge gain of 19.04%, followed by Accra with 16.0%.

**Figure 5:**
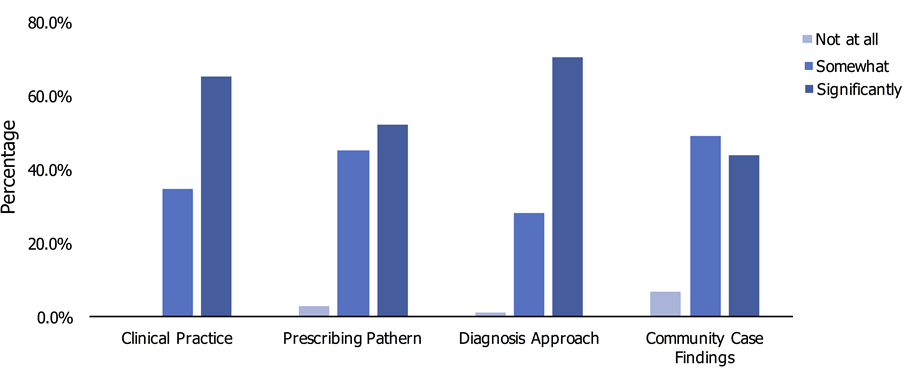
Impact of M-PACT training on clinical practice, prescription pattern, diagnostic approach and community-based case finding among participants, 2015

A 6-months' post-training review of participants revealed that there was an influence on knowledge and clinical practice in 100% of participants, which was significant in 65.28%; with over 97% (significantly in 52.11%) on prescription patterns; over 98% on diagnostic approaches (significantly in 70.42%); and over 91% on case findings in the community (significantly in 43.66%) (Figure 5). Furthermore, we discovered that a number of participants cascaded their training and expereince to other physicians as they had gone back to their localities and held educational sessions with doctors from surrounding satellite institutions on better management and monitoring of TB and HIV. Some consulted and still consult in primary health care centers as they were able to prescribe more confidently, especially with respect to pediatric prescriptions. One participant was able to implement testing for malaria parasites using the Rapid Diagnostic Test (RDT) before prescribing drugs. Both participants and faculty were highly impressed with the organization and content of all the courses with over 90% in all course participants grading their individual courses as either very good or excellent in the final evaluation. Participants thoroughly enjoyed the few topics on health leadership and management and mentoring. Most participants requested that leadership and mentoring should be institutionalized and made available to all doctors. A Freetown participant said, “I thoroughly enjoyed this course and would like to recommend that other topics in internal medicine be considered in further programs. The course should be organized again in Sierra Leone, so that other medical doctors will benefit.” Another said, “The lecturers were very helpful. I appreciated the mentoring sessions, the interaction with colleagues and networking with others. It was very helpful.” In Dakar, the course organizers, has this to say, “All the sessions went well and the objectives reached. The realities of the field visits provoked thoughts on what strategies to put in place”

**Learning points**: Positive feedback from participants showed the immediate impact of the course. This was further strengthened by positive influence on clinical practice, prescription patterns and diagnostic approaches of participants and case finding in the community. These impacts are multiplied by participant-led trainings cascaded to other cadres of healthcare workers. Also, the improved percentage of trainees who successfully passed their Membership examination from the WACP validated these claims. However, from the 3-year proof-of-concept program, we learned the following: training should be adaptable and tailored to solve identified problems within the population of the trainees and those they serve. This was seen with the inclusion of outbreak investigations following the Ebola Viral Disease outbreak in West Africa (2014-2016); appropriate trainees should be selected for relevant courses. In this course, participants were selected from a pool of qualified doctors at all levels of care. Selection was open and followed a defined protocol; training should be facilitated by skilled and competent individuals. Skilled and experienced physicians were used throughout in the delivery of this course. Despite their experiences and skills, the WACP faculty was, however, first subjected to the Doctors as Educators (DAE) training to refresh their delivery skills and knowledge before participating in the course; North-South collaborations and twinning should be encouraged: The North-South Collaboration and later South-South Collaboration worked so well in this project with faculty from United Kingdom participating in most of the courses in Nigeria, Ghana, Senegal and Sierra Leone [6]. The later involvement of skilled faculty from Nigeria in Sierra Leone training exemplified South-South collaboration. Both models were very successful and should be encouraged in global health; trainees should be encouraged and supported to train others. As training others improves understanding of concepts and develops new skilled human resources for health and delay knowledge decline, this should be encouraged. All alumni should be encouraged to send in evidence of cascade training to more junior staff and allied healthcare professionals.

## Discussion

The program demonstrated its effectiveness as there was an immediate impact illustrated by positive participant feedback. Evaluation of the impact of the first six courses showed a positive influence on the clinical practice of participants on prescription patterns, diagnostic approaches and case finding in the community [7]. Over the past 3 years there has been a decline in incidence and mortality due to malaria, HIV and TB, to which this training has contributed. The results (Figure 4) showed a progressive increase in knowledge gained which could be linked to the implementation of feedback obtained from previous courses, as well as improved delivery capacity of the faculty. The differences in pre-course clinical knowledge scores and knowledge gain from the courses were largely due to the different backgrounds and prior experiences of physicians attending in different locations. However, each of the courses made a significant impact overall on subsequent clinical practice, as demonstrated by the 6-month post-training review (Figure 5). The slight decrease noticed in the fifth course (Figure 4) could be attributed to the fact that the course was wholly delivered by local faculty and that was their first delivery of a full M-PACT course without RCP volunteers. Repeating the process will most likely result in higher knowledge gain from improved delivery capacities. Following training, there has been an improvement in the number of trainees attaining their Membership exam from the WACP (from current examination pass rate and participants' reports). In turn, this is highly motivational and will result in a change in the delivery of care, resulting in improved patient outcomes. There have been continual requests in all regions for more courses from positive feedback to colleagues and the dissemination of information. Physicians are now better trained to react in a practical and effective manner to contain future infectious disease outbreaks. In order to keep up the momentum gained through this postgraduate education program, the WACP should take the lead in securing public-private financing for healthcare training, as was demonstrated effectively by the relationship with the Eco Bank Foundation. Many companies in Africa implement Corporate Social Responsibility programs which operate for the common good and such corporations may not necessarily be operating in the healthcare sector. While pharmaceutical companies are traditionally thought of as a potential source of funding for healthcare and educational schemes (and indeed, Johnson and Johnson, GSK, Gilead, Novartis and Abbott are good examples of these), medical leaders need to think outside the box, approaching all commercial sectors from finance, petrochemical industries and mining to modern technology, such as internet and mobile phone companies. It would seem that only arms dealers, cigarette and alcohol companies are off bounds in this regard. With tight budgets in Africa, we should look to countries whose companies gain large profits from Africa, such as China, Europe and India to be forthcoming in their corporate social responsibility, so that what has been gained is not lost.

## Conclusion

The joint partnership between RCP and WACP to organise in-service training courses in five training centers in West Africa over the 3-year period, evaluated based on participant feedback and WACP Membership exam performance, was successful. This proof-of-concept program demonstrates the feasibility and the need for continuing and further developing this public-private partnership model for in-service training of healthworkers.

### What is known about this topic

- Continuous professional development training is important to sustain the competency of healthcare workers and improve healthcare servies;
- In-service training represents a significant cost to the government so that alternative funding mechanisms had to be sought.

### What this study adds

- The 3-year proof-of-concept training program organised by a collaboration between RCP and WACP demonstrated the feasibility and benefits of in-service training;
- North-South and South-South collaboration in healthcare training is fruitful and should be encouraged in global health;
- Ensuring the leadership and communication skills of the educators is essential to the outcome of the training.

## Competing interests

The authors declare no competing interest.
